# Molecular Mechanisms of Synergistic Effect of PRIMA‐1^met^ and Oxaliplatin in Colorectal Cancer With Different p53 Status

**DOI:** 10.1002/cam4.70530

**Published:** 2025-01-05

**Authors:** Xiao‐lan Li, Jianbiao Zhou, Nicole Xin‐Ning Tang, Yi Chai, Meng Zhou, Ai‐di Gao, Zhong‐kai Lu, Han Min

**Affiliations:** ^1^ Department of Gastroenterology The Affiliated Suzhou Hospital of Nanjing Medical University, Suzhou Municipal Hospital Suzhou Jiangsu People's Republic of China; ^2^ Cancer Science Institute of Singapore National University of Singapore Singapore; ^3^ Department of Medicine, Yong Loo Lin School of Medicine National University of Singapore Singapore; ^4^ NUS Centre for Cancer Research, National University of Singapore Singapore; ^5^ Changzhou No. 4 People's Hospital Changzhou City Jiangsu Province People's Republic of China

**Keywords:** colorectal cancer (CRC), combination therapy, drug resistance, hematologic toxicity, oxaliplatin (L‐OHP), p53 tumor suppressor gene, PRIMA‐1^met^

## Abstract

**Background:**

The toxicity and drug resistance associated with oxaliplatin (L‐OHP) limit its long‐term use for colorectal cancer (CRC) patients. p53 mutation is a common genetic trait of CRC. PRIMA‐1^met^ (APR‐246, eprenetapopt) restores the DNA‐binding capacity of different mutant P53 proteins. PRIMA‐1^met^ has progressed to the Phase III clinical trial. Our study explores the combination therapy of PRIMA‐1^met^ and L‐OHP for CRC with different p53 status.

**Methods:**

Cell viability was assessed with Cell Counting Kit‐8 (CCK‐8) assay and combination index (CI) was calculated using The Chou‐Talalay method. We also employed wound healing assay and colony formation assay to determine the effect of L‐OHP, PRIMA‐1^met^ and their combination. Weighted gene co‐expression network analysis (WGCNA) of RNA‐seq data was conducted to identify key modules and central genes related to different treatment modalities. Xenograft CRC mouse model was used to assess the combination treatment in vivo.

**Results:**

Our findings showed heightened cytotoxicity and inhibition of migration, and colony formation in CRC cells treated with both drugs, irrespective of p53 status, presenting a promising avenue for addressing L‐OHP resistance and toxicity. RNA‐seq analysis revealed differential responses between p53‐wide type HCT116 and p53‐mutant DLD‐1 cells, with pathway alterations implicated in tumorigenesis. WGCNA identified key modules and hub genes associated with combination therapy response. In vivo studies demonstrated enhanced efficacy of combined therapy over PRIMA‐1^met^ alone, while mitigating L‐OHP‐induced toxicity.

**Conclusions:**

In summary, our research reveals the differential molecular mechanisms of combined PRIMA‐1^met^ and L‐OHP in CRC with wild type p53 and mutant p53. Our data not only demonstrate that this combined regimen exerts synergistic anti‐CRC effect in vitro and in vivo, but also suggest the benefit of PRIMA‐1^met^ on prevention of L‐OHP‐related side effects. These findings underscore the clinical potential of PRIMA‐1^met^‐L‐OHP combination therapy in CRC, offering enhanced efficacy and reduced toxicity, warranting further clinical investigation.

## Graphical abstract

We report differential molecular mechanisms including pathways, key modules, and hub genes, induced by combination of oxaliplatin (L‐OHP) and PRIMA‐1^met^ (APR‐246, eprenetapopt) in p53‐wild type vs. p53‐mutant colorectal cancer. Our in vivo studies show that the additional of PRIMA‐1^met^ offers a threefold advantage: enhanced therapeutic effects, reduced L‐OHP resistance, and prevention of L‐OHP‐related side effects.

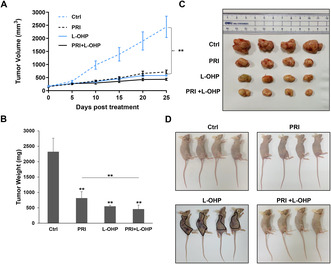

**Abbreviations**
ANOVA: one‐way analysis of variance
ARRIVE: Animal Research: Reporting of In Vivo Experiments
CCK‐8: Cell Counting Kit‐8
CI: combination index
CRC: colorectal cancer
DEGs: differentially expressed genes
DMEM: Dulbecco's modified Eagle's medium
DMSO: dimethyl sulfoxide
FDR: false discovery rate
FOLFOX: a combination of 5‐fluorouracil (5‐FU), oxaliplatin (L‐OHP) and calcium folinate (CF)
GEO: Gene Expression Omnibus
L‐OHP or Oxa: oxaliplatin
PRIMA‐1: p53 reactivation and induction of massive apoptosis‐1
PRIMA‐1^met^
: PRI, APR‐246, eprenetapopt
TV: tumor volume
WGCNA: weighted gene co‐expression network analysis

## 1 Background

The global impact of colorectal cancer (CRC) is significant, with approximately 1.9 million new cases and 0.9 million fatalities reported in 2020. This positions CRC as the third leading cause of cancer‐related deaths according to the World Health Organization's Cancer Fact Sheet. The dissemination of CRC to prevalent sites such as the liver, lungs, and peritoneum is a key factor contributing to its elevated mortality rate [1]. Unfortunately, 20% of CRC patients are diagnosed with metastasis from the outset, and around 70% of patients will eventually experience a relapse with metastatic progression [1]. Despite a notable improvement in the 5‐year survival rate for late‐stage CRC over the past two decades, rising from 16% to 26%, it still lags significantly behind the 90% survival rate observed in cases diagnosed at an early stage [2, 3]. The FOLFOX regimen, a combination of 5‐fluorouracil (5‐FU), oxaliplatin (L‐OHP, Oxa) and calcium folinate (CF), is commonly used as the first‐line therapy for CRC patients at Stages III and IV [4, 5, 6]. L‐OHP, a pivotal platinum therapeutic agent, demonstrates efficacy in treating solid tumors, especially digestive cancers [7]. Its cytotoxic impact primarily stems from inducing DNA damage and inhibiting DNA replication, resulting in cell cycle arrest and apoptosis [8, 9]. Nevertheless, the challenge of drug resistance to L‐OHP frequently hinders the effective treatment of CRC patients, and mortality is linked to both metastasis and resistance to chemotherapy. Consequently, there is a pressing requirement to devise innovative therapeutic strategies, including combination therapies with novel targeted agents or Traditional Chinese Medicine (TCM) [10], to surmount L‐OHP resistance.

Functioning as a stress‐responsive transcription factor, p53 plays a crucial role in regulating various biological processes, including cell cycle arrest, DNA repair, apoptosis, autophagy, and senescence [11]. Notably, p53 mutation occurs in 40%–50% of CRC cases, resulting in the loss of its tumor‐suppressive function. This loss contributes to the progression of adenoma to carcinoma during colorectal carcinogenesis [12, 13]. The presence of p53 mutations or deletions is linked to an elevated risk of tumor recurrence, metastasis, and increased mortality in CRC patients [14, 15, 16].

Mutations in p53 also contribute to heightened resistance to L‐OHP by negating the DNA damage response induced by L‐OHP in CRC cells. This response relies on activated p53 signaling for apoptosis and cell cycle arrest [17, 18]. Consequently, reactivating the compromised p53 signaling emerges as a promising strategy to overcome L‐OHP resistance [19, 20]. PRIMA‐1^met^ (PRI, APR‐246, eprenetapopt) stands out as a methylated analogue of PRIMA‐1 (p53 reactivation and induction of massive apoptosis‐1), capable of restoring the specific DNA‐binding region of mutated p53 and reinstating its wild‐type (wt) function [21, 22]. In prior research, we documented that PRIMA‐1^met^ inhibited the growth of CRC cells independently of p53 status. Notably, it selectively induced apoptosis in CRC cell lines with mutant p53 by upregulating the pro‐apoptotic protein Noxa [23]. The synergistic anti‐tumor properties of PRIMA‐1/PRIMA‐1^met^ have been observed in combination with chemotherapy, radiotherapy, and targeted therapy across various cancer types, including colon, head and neck, lung, esophageal, ovarian, and pancreatic cancers, as well as chronic lymphocytic leukemia (CLL) and acute myeloid leukemia (AML) [22, 24, 25, 26, 27, 28, 29].

In this study, we aim to investigate the biological impact of combining PRIMA‐1^met^ with L‐OHP in CRC cells possessing either wt or mutant p53 and their underlying molecular mechanisms. Additionally, the study aims to examine the sensitizing effect of PRIMA‐1^met^ on L‐OHP resistance both in vitro and in vivo.

## 2 Methods

### 2.1 Cell Lines and Drugs

A panel of 5 colorectal cancer cell lines with different p53 statuses was used in this study including HCT116 (p53‐wt), RKO (p53‐wt), HCT15 (mutant p53‐P153A), SW620 (mutant p53‐R237H), and DLD‐1 (mutant p53‐S241F). These cell lines were purchased from Shanghai Cell Bank of Chinese Academy of Sciences (Shanghai, China). DLD‐1 and HCT15 cells were cultivated in RPMI‐1640 medium (HyClone, Logan, USA), SW620 cells were cultivated in Leibovitz's L15 medium (Gibco Life Technology, New York, USA), HCT116 cells were cultivated in McCoy's 5A medium (Gibco Life Technology, New York, USA) and RKO cells were cultivated in DMEM (Dulbecco's modified Eagle's medium) (HyClone, Logan, USA). All culture media were supplemented with 10% inactivated foetal bovine serum (FBS, Gibco Life Technology, New York, USA) and 1% penicillin/streptomycin (Invitrogen, Carlsbad, CA). Cells were cultured in a humid incubator with 5% CO_2_ at 37°C. PRIMA‐1^met^ (Santa Cruz Biotechnology, Santa Cruz, CA) and L‐OHP (Sigma‐Aldrich, St. Louis, USA) were dissolved in DMSO (dimethyl sulfoxide) at a concentration of 50 mM, stored at −20°C, and diluted to appropriate concentrations in cell culture medium for experiments. The same volume of DMSO was used in experiments as a control.

### 2.2 Cell Counting Kit‐8 Proliferation Assay

CRC cells were seeded in a 96‐well plate at a density of 7000 cells/well in 100 μL medium. After 24 h, cells were treated with PRIMA‐1^met^ and L‐OHP at fixed ratios of 1:0.2 (HCT116), 1:0.6 (RKO), 1:2 (HCT15), 1:2 (SW620), and 1:1.3 (DLD‐1) and incubated for 48 h. Cell Counting Kit‐8 (CCK‐8) proliferation assays (Dojindo, Shanghai, China) were performed to test cell proliferation via WST‐8 formazan dye quantification, which gives the proportion of living cells. DMSO samples served as a control, and their value was set as 100%. The values of other samples were calculated and presented as percentages relative to the DMSO controls. IC_50_ values were determined by CCK‐8 assay and calculated by CalcuSyn software (Biosoft, Cambridge, UK). Each experiment was performed in triplicate.

### 2.3 Combination Index and Isobologram Analysis

The Chou‐Talalay method [30] was applied to study combination effects of PRIMA‐1^met^ and L‐OHP from their cell proliferation data at different concentrations. The median‐effect equation provided the theoretical basis for the combination index (CI)‐isobologram equation for the quantitative determination of PRIMA‐1^met^ and L‐OHP interactions. CalcuSyn (Biosoft, Cambridge, UK) was used to calculate CI index and construct isobolograms. The evaluation criteria of drug interactions were according to the CI values proposed by Chou‐Talalay, offering quantitative definition for additive effect (CI = 1), synergism (CI < 1), and antagonism (CI > 1) in drug combinations as described before [31].

### 2.4 Wound Healing and Transwell Invasion Assay

RKO and DLD‐1 cells were seeded into 6‐well plates at a density of 8 × 10^5^ cells/well. After 48 h, the cells were cultured to near confluence, and a wound was scratched through the center of the well. Then, the cells were gently rinsed with PBS and replaced with 2 mL low‐serum medium containing additives (DMSO, PRIMA‐1^met^, L‐OHP or the combination). Pictures of the scratches were taken under a microscope (Olympus, Japan) at 10× magnification at 0 and 48 h from the same field for each treated sample and on the same color channel for each cell line. The areas of each scratch were calculated using ImageJ version 1.8.0 (National Institutes of Health, USA) for comparisons. The invasiveness of cells was evaluated with an invasion assay using transwell chambers (Corning, NY, USA) with 8‐μm pore‐sized filter. Matrigel basement membrane matrix (Corning, 100 μg/mL, 15 μL/well) was added to each well before cells were plated on the upper chamber and incubated at 37°C for 30 min. The DLD‐1 and RKO cells (1 × 10^5^ cells/well per line) were placed in upper‐chamber inserts with a serum‐free medium containing additive (DMSO, PRIMA‐1^met^, L‐OHP or the combination), while the bottom chamber contained complete medium containing 10% FBS. After 24 h, the invaded cells were fixed with 4% formaldehyde and stained with 0.05% crystal violet solution (Merck, NJ, USA) following the removal non‐invaded cells. Invaded cells that passed through the pores were calculated in three randomly selected areas under a microscope (Olympus, Japan). All invaded cells in each chamber insert were dissolved with 33% acetic acid and assessed at 570 nm using a Tecan Spark plate reader (Tecan, Switzerland). Each experiment was repeated three times.

### 2.5 Colony Formation Assay

HCT116 and DLD‐1 cells were plated at 5000 cells/well on 12‐well plates in triplicates in soft agar (0.6% agar for base‐layer, 0.44% agar for top‐layer with cells). A further 500 μL of 1× culture medium with DMSO, PRIMA‐1^met^, L‐OHP or the combination at indicated doses was added onto the top‐layer. Plates were maintained at 37°C for 14 days with exchanging the media and fresh drugs for twice per week. At the end of incubation, the culture medium was replaced with 1 × PBS. Colonies, defined as at least eight cells in one cluster, were counted under the white field of a Nikon Stereo Microscope SMZ1270I (Nikon Corporation, Japan).

### 2.6 RNA‐Seq

HCT116 (p53‐wt) and DLD‐1 (p53‐mutant) cells were treated with either DMSO or PRIMA‐1^met^ (45 μM for HCT116; 20 μM for DLD‐1) or L‐OHP (6 μM for HCT116; 35 μM for DLD‐1) or combination of these two drugs for 48 h. Each treatment was performed in triplicates. Total RNA was extracted using the RNeasy mini kit (Qiagen). RNA quantity, quality, and purity were assessed with the use of the RNA 6000 Nano assay on the Agilent 2100 Bioanalyzer (Agilent Technologies, Santa Clara, CA). RNA sequencing (RNA‐seq) libraries were constructed by TruSeq Library Prep Kit (Illumina, San Diego, CA) according to the manufacturer's instructions and subjected to Illumina Novaseq deep sequencing (paired‐end reads of 150 bases) at BGI (Shenzheng, China). The RNA‐seq data were checked for raw sequence quality using FastQC v0.11.5, and were filtered to remove adaptor sequences, contamination and low quality read. The sequences were mapped to human genome hg38 using STAR v2.4.2a and subsequently, transcript quantification using RSEM 1.2.25 with Gencode v24 annotation as described previously [32]. These raw RNA‐seq data have been deposited into the Gene Expression Omnibus (GEO) repository with the accession number GSE254323.

### 2.7 Differential Expression and Pathway Analysis of RNA‐Seq Data

Gene expression in CRC cells treated with single agent or the combination was compared to DMSO‐treated samples as baseline. To identify and analyze differentially expressed genes (DEGs), we employed the DESeq2 package in R. DEGs were discerned using a false discovery rate (FDR) threshold of < 0.05 and an absolute log2 fold change (|log2FC|) ≥ 1. Heatmaps illustrating the top significant DEGs were created using the pheatmap package in R, with genes and samples clustered through complete linkage and Euclidean distance. For pathway enrichment analysis of the identified DEGs, the clusterProfiler package was utilized, maintaining a significance threshold of *p* ≤ 0.05 for both KEGG and GO enrichment analyses.

### 2.8 Immunoblotting Assay

After indicated treatment for 48 h, HCT116 cells were lysed in RIPA lysis and extraction buffer (Thermo Fisher Scientific), supplemented with proteinase inhibitor cocktail and phosphatase inhibitor cocktail (Roche) for 30 min on ice. Bradford assay was used to determine protein concentration. Immunoblotting was performed using SDS‐PAGE followed by protein transfer to PVDF membrane (Bio‐Rad). The following antibodies were used: β‐catenin (1:1000, Cell Signaling Technology, CST, #9582); Phospho‐β‐catenin (Ser33/37/Thr41) (1:1000, CST, #9561); CTGF (1:1000, CST, #86641), YAP (1:1000, CST, #14074). Primary antibodies were incubated overnight in cold room. Secondary antibodies were incubated for 1 h at room temperature. β‐actin (1:5000, Santa Cruz Biotechnology, sc‐47778) was used as loading control; The signals were detected with SuperSignal reagents (Thermo Fisher Scientific) on a ChemiDoc MP Imaging System (Biorad).

### 2.9 WGCNA Analysis and Key Module Identification

Weighted gene co‐expression network analysis (WGCNA) was applied to identify key modules associated with different treatment conditions. Optimal soft‐thresholding power (*β* = 6 for HCT116 and 5 for DLD‐1) was determined to construct a scale‐free network, followed by dynamic tree cutting for module detection. The significance of each module (MS) was evaluated based on the average absolute gene significance (GS) of all genes within the module, with GS quantified as the log10 transformation of the *p*‐value in linear regression between gene expression and clinical traits. Modules with the highest MS were considered key modules for further analysis. Key modules are exported to visualize in cytoscape with weight cutoff = 0.5. Hub genes are prioritized as top 10 genes with highest degree of connection in CytoHubba algorithms with cutoff = top 10; weight threshold = 0.8.

### 2.10 Xenograft CRC Mouse Model

All animal experiments were complied with the Animal Research: Reporting of In Vivo Experiments (ARRIVE) guidelines [33]. Female BALB/c mice (18–20 g, 4–6 weeks old) were purchased from Shanghai SLAC Laboratory Animal Company (Shanghai, China) and housed in specific pathogen‐free conditions. Each group consisted of 4 randomly assigned mice. Exponentially growing DLD‐1 cells (4 × 10^6^) were subcutaneously injected into loose skin in the right inguinal region of recipient mice. Treatment was started after 12 days, when the mean tumor volume was approximately 165 mm^3^. Mice were treated with PRIMA‐1^met^ at 40 mg/kg/day, or with L‐OHP at 5 mg/kg twice a week by intraperitoneal (I.P.) injection or with their combination. Mice in the control group were treated with an equal volume of PBS daily. The length (L) and width (W) of tumors were measured with calipers. The tumor volume (TV) was calculated as (L × W^2^)/2. At day 25 post‐treatment, mice were euthanized, and tumors were removed for imaging. The tumor weight was measured with an electronic scale. All the animal procedures were approved by the Animal Ethics Committee of Nanjing Medical University (protocol number: 2018‐036).

### 2.11 Statistical Analysis

The statistical analysis was performed with SPSS version 19.0. Values are expressed as the mean ± standard error of the mean (SEM). Comparisons between groups were made using one‐way analysis of variance (ANOVA). Tumor volume and weight reduction were compared by Student's *t*‐test. *p* < 0.05 was considered to be statistically significant.

## 3 Results

### 3.1 PRIMA‐1^met^ and L‐OHP Synergistically Inhibited CRC Cell Proliferation

**FIGURE 1:**
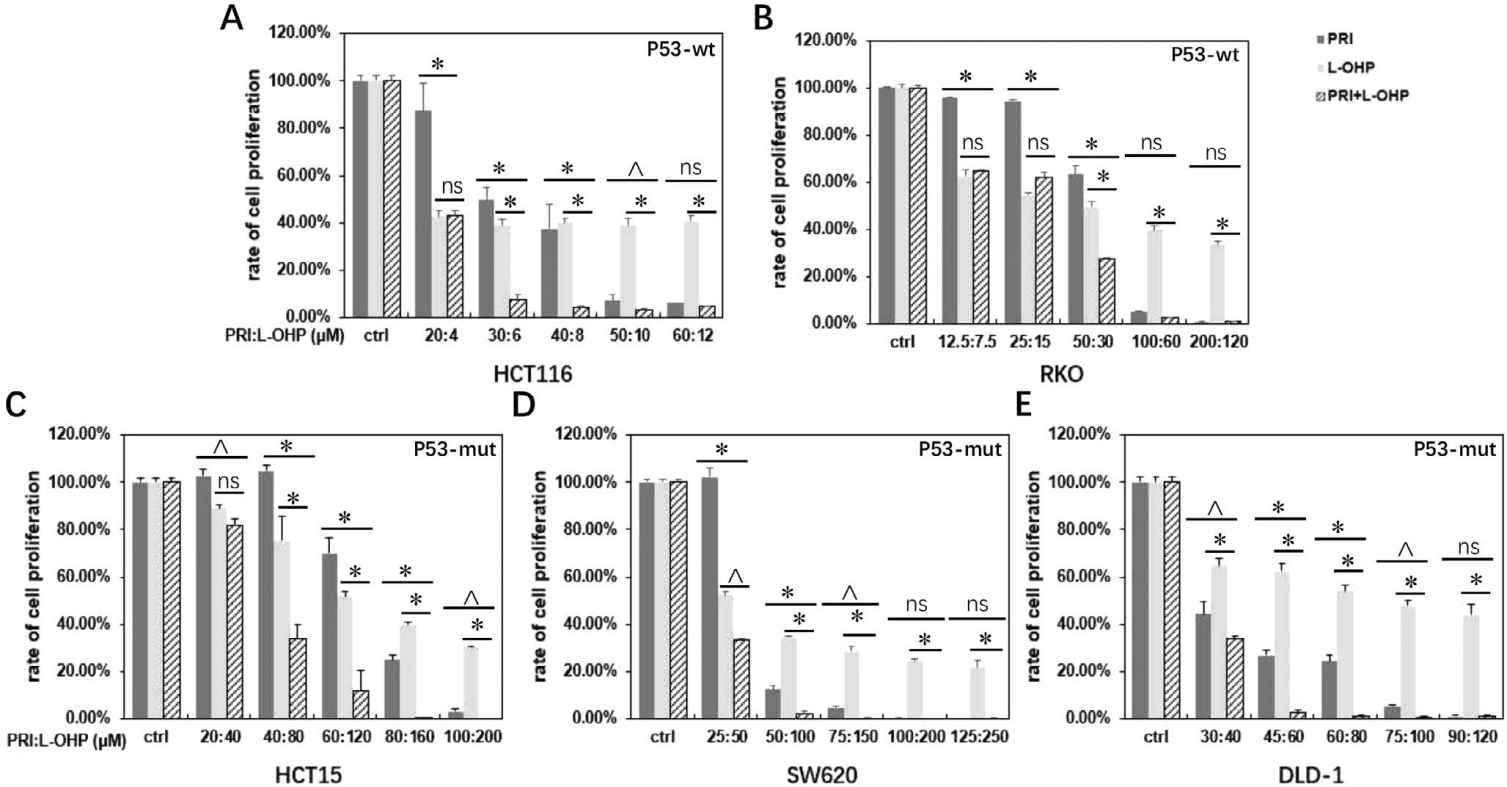
The inhibitory effects of PRIMA‐1^met^ (PRI) alone, L‐OHP alone and their combinations on CRC cell lines. The combination of PRI and L‐OHP at the indicated ratios of 1:0.2 for HCT116 (A), 1:0.6 for RKO (B), 1:2 for HCT15 (C), 1:2 for SW620 (D), and 1:1.3 for DLD‐1 (E) enhanced cytotoxicity in CRC cells with different p53 statuses compared to either of single drug treatment. Following 48 h of treatment with the two drugs, cell proliferation was estimated using the CCK‐8 proliferation assay, and the percentage was normalized to the DMSO control (ctrl). Each experiment was performed in triplicate, and error bars represent the standard error of the mean (SEM). Black lines indicate the comparison between combination treatment vs. PRI alone or L‐OHP alone. *p < 0.01; ^*p* < 0.05; ns; not significant. wt, wild type; mut, mutant.

We first evaluated the inhibitory effects of PRIMA‐1^met^, L‐OHP or their combination on the panel of 5 distinct CRC cell lines with different p53 status. After a 48‐h treatment with either PRIMA‐1^met^ or L‐OHP individually, there was a dose‐dependent reduction in cell proliferation observed in HCT116 (Figure 1A), RKO (Figure 1B), HCT15 (Figure 1C), SW620 (Figure 1D), and DLD‐1 (Figure 1E) cells. Combining PRIMA‐1^met^ with L‐OHP exhibited a more pronounced suppression compared to each individual agent, and this effect was predominantly concentration‐dependent (Figure 1A–E).

**FIGURE 2:**
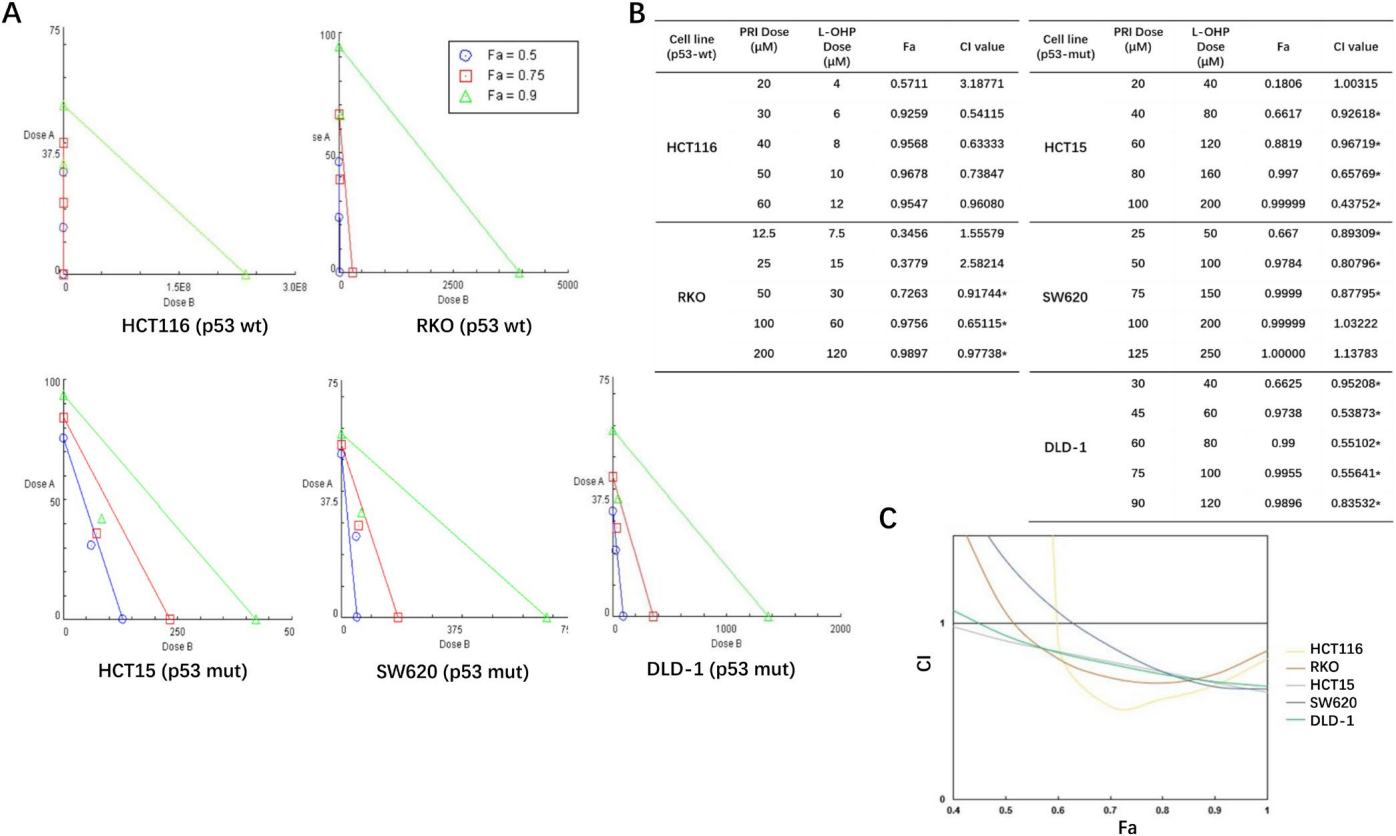
PRIMA‐1^met^ (PRI) and L‐OHP synergistically inhibited cell growth in different CRC cell lines. (A) Isobologram analysis of the combination of PRI and L‐OHP in RKO, HCT, SW620, and DLD‐1. The individual doses of PRI and L‐OHP to achieve 90% (green line) growth inhibition (Fa = 0.90), 75% (red line) growth inhibition (Fa = 0.75), and 50% (blue line) growth inhibition (Fa = 0.50) were plotted on the *x*‐and *y*‐axes. CI values calculated using Calcusyn software is represented by points above (indicate antagonism between drugs) or below the lines (indicate synergy). Drug A is PRI and drug B is L‐OHP). (B) The combination index (CI)‐fraction affected (FA) curves for CRC cells exposed to PRI and L‐OHP in a fixed molar ratio based on the IC_50_. Drug exposure was 48 h. (C) This plot was fit by the program based on the actual values, showing synergism (CI < 1) at high FA values.

Analysis of CI and fraction affected (Fa) values revealed a synergistic effect between the two drugs (Figure 2A,B), with the co‐treatment leading to a reduction in cell proliferation by over 66% across all tested CRC cell lines (Figure 2B). Notably, the highest synergism between PRIMA‐1^met^ and L‐OHP was observed at elevated concentrations, with Fa values approaching 1.0 in the three p53‐mutant cell lines, including HCT15, SW620, and DLD‐1, suggesting near‐complete inhibition (Figure 2C). In the two wt p53 lines, HCT116 and RKO, the Fa value was approximately 0.75 (Figure 2C). Interestingly, p53‐mutant cells exhibited relatively lower sensitivity to L‐OHP monotherapy, with their IC_50_ values being 2‐ to 20‐fold higher compared to those expressing wt p53 (Table 1). These findings align with the understanding that mutant p53 significantly contributes to resistance against L‐OHP [34].

**TABLE 1:** IC_50_ values of PRIMA‐1^met^ and oxaliplatin in different CRC cell lines.

Cell line	p53 status	IC_50_	
		PRIMA‐1^met^ (μM)	Oxaliplatin (μM)
HCT116	Wild‐type	31	0.029
RKO	Wild‐type	46	24
HCT15	Mutant, P153A	76	130
SW620	Mutant, R237H	50	52
DLD‐1	Mutant, S241F	33	93

Taken together, our results underscore the capacity of PRIMA‐1^met^ to synergistically interact with L‐OHP against CRC cells, effectively resensitizing L‐OHP‐resistant p53‐mutant CRC cells to L‐OHP.

### 3.2 The Combination of PRIMA‐1^met^ and L‐OHP Suppressed CRC Cell Migration and Invasion

**FIGURE 3:**
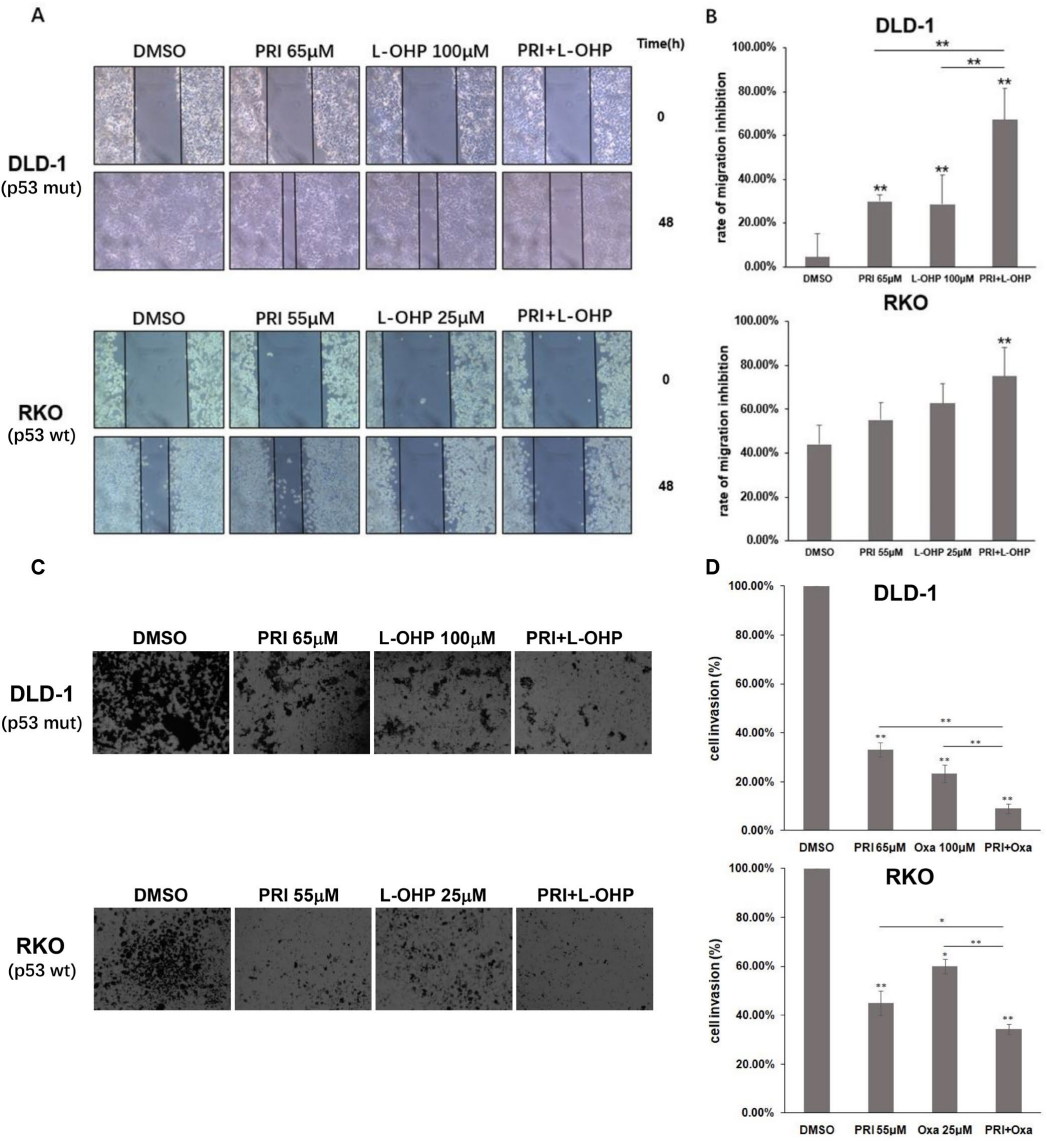
Effects of PRIMA‐1^met^ (PRI), L‐OHP and co‐treatment of PRI and L‐OHP on CRC cell migration and invasion. (A) A wound healing assay showed that the migration and invasion ability of DLD‐1 (p53‐mutant) and RKO (p53‐wt) cells were robustly inhibited by the co‐treatment of PRI and L‐OHP. Cell wounds were observed, and pictures were taken under a microscope (Olympus Japan) at 10× magnification from the same field for each treated sample as indicated at 48 h after the scratching. Two black lines showed the size of the remaining wound in each image. (B) Cell wounds were measured and quantified with ImageJ program (National Institutes of Health, USA). Data are presented as the mean ± SEM of three independent experiments Asterisk** represents statistically significant difference of *p* < 0.01. (C) Through transwell assay, DLD‐1 and RKO cells were treated with DMSO, PRI, and the combination at indicated does for 24 h. Then, the number of trans‐membrane cells to the lower chamber was detected. The representative images were shown (magnification: 10 × 10). (D) The relative migration rate was determined. Data are presented as mean ± SEM, *n* = 3. *p < 0.05, **p < 0.01.

Tumor metastasis is a complex process and contains a series of requirements, including infiltration of cancer cells into the primary site (invasion) and migration to other sites via the blood and lymphatic systems [35]. To examine the effect of the cotreatment on cell migration and invasion, a wound healing assay and a transwell invasion assay were performed on DLD‐1 (p53‐wt) and RKO (p53‐mutant) cells after treatment with DMSO, PRIMA‐1^met^, L‐OHP and the two‐drug combination for 48 and 24 h, respectively. As shown in Figure 3A,B, scratch recovery was found to be significantly suppressed after combined treatment with PRIMA‐1^met^ and L‐OHP in both DLD‐1 and RKO cells compared with that in cells treated with the DMSO control (*p* < 0.01). Although both of the two drugs alone decreased the number of DLD‐1 cells presented in the scratch, the combination was superior to either single agent (*p* < 0.01). We further loaded Matrigel in transwell chambers to assess whether PRIMA‐1^met^ can enhance the anti‐invasion effects of L‐OHP. As shown in Figure 3C,D, treatment with PRIMA‐1^met^ or L‐OHP alone significantly decreased cell invasion in DLD‐1 and RKO cells. However, the combination of PRIMA‐1^met^ and L‐OHP further augmented the inhibitory effect, reducing cell invasion by 91% in DLD‐1 cells and 66% in RKO cells (*p* < 0.01). Taken together, these results suggest that PRIMA‐1^met^ and L‐OHP synergistically inhibited motility and invasion signaling in CRC cells, regardless of p53 status.

### 3.3 PRIMA‐1^met^ Enhanced L‐OHP‐Mediated Inhibition of Colony Formation in CRC Cells

**FIGURE 4:**
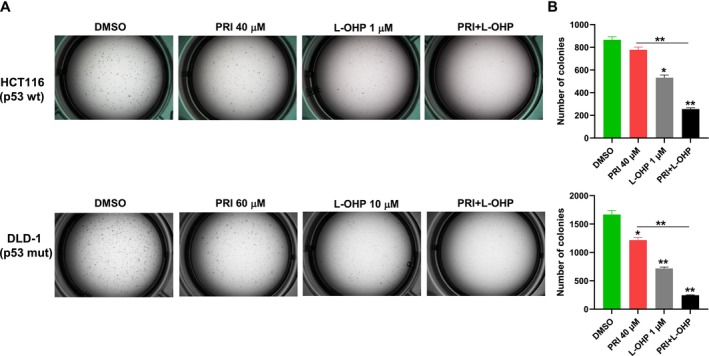
Clonogenic assay showed the long‐term effects of combination treatment of CRC cells with PRIMA‐1^met^ (PRI) and L‐OHP. (A) HCT116 (upper panel) and DLD‐1 (lower panel) cells were seeded in soft agar in 12‐well plates and. Culture medium containing DMSO, PRIMA‐1^met^, L‐OHP or the combination at indicated doses were replaced twice per week for 2 weeks. A photograph of each well in a representative experiment was shown. (B) Cell colonies comprising more than 8 cells were counted under a low‐magnification microscope (Nikon Corporation, Japan). Data were shown from three independent experiments, and the error bars represented the SEM. *p < 0.05; **p < 0.01.

The clonogenic assay, a widely utilized method for evaluating the capability of individual cancer cells to generate progeny in vitro, also mirrors the morphological transformations observed in vivo [36, 37]. In our exploration of the suppressive effects of the PRIMA‐1met–L‐OHP combination on malignant transformation, the activity of the single drug or the combination was analyzed in in vitro tumor formation assays with HCT116 (p53‐wt) and DLD‐1 (p53‐mutant) cells growing in soft agar. The cells were subjected to PRIMA‐1^met^‐containing, L‐OHP‐containing or combination regimens that were repeated twice per week for 2 weeks. The administration of PRIMA‐1^met^ as single agent led to a significant reduction in the number of colonies of DLD‐1 (p53‐mutant), but not HCT116 (p53‐wt) compared to their cells treated with the DMSO control (*p* < 0.05 for DLD‐1 cells). Treatment with L‐OHP alone significantly suppressed the colony forming capacity of HCT116 and DLD‐1 cells (*p* < 0.05 for HCT116 cells; *p* < 0.01 for DLD‐1 cells). But simultaneous PRIMA‐1^met^ plus L‐OHP plus oxaliplatin resulted in further increased activity as a more potent inhibition of colony formation than either of single‐agent in both of cell lines was documented (*p* < 0.01 for all comparisons for both HCT116 and DLD‐1) (Figure 4A,B). In summary, these data demonstrate that PRIMA‐1^met^ can synergize with L‐OHP to reduce clonogenic potential of CRC cells, independent of their p53 status.

### 3.4 GO Function and KEGG Pathway Analyses of DEGs From PRIMA‐1^met^, L‐OHP or Co‐Treatment

**FIGURE 5:**
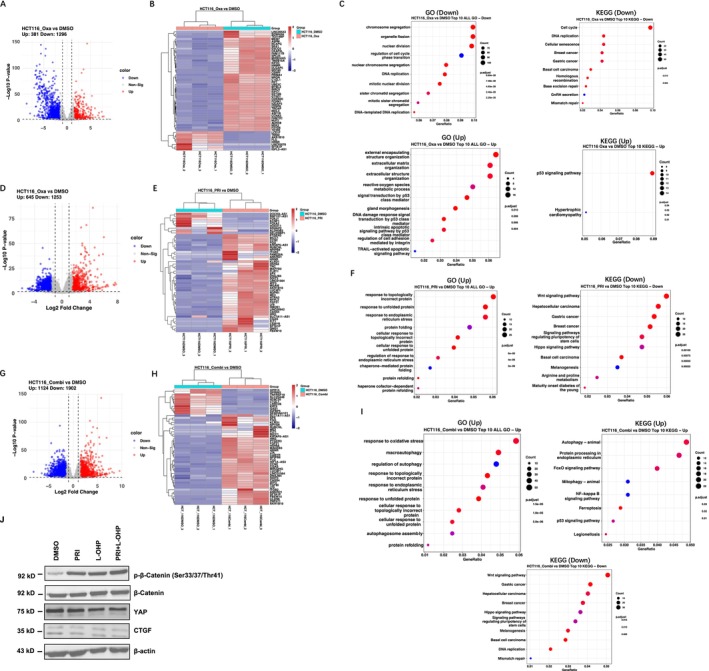
Analysis of DEGs in p53‐wt HCT116 treated with different modalities. (A) Volcano plot of all significantly changed and (B) heatmap of top 50 DEGs ranked by fold change in L‐OHP vs. DMSO. (C) GO and KEGG pathway analysis of DEGs in group of L‐OHP vs. DMSO. (D) Volcano plot of all significantly changed and (E) heatmap of top 50 DEGs ranked by fold change in PRI vs. DMSO. (F) GO and KEGG pathway analysis of DEGs in group of PRI vs. DMSO. (G) Volcano plot of all significantly changed and (H) heatmap of DEGs screening between combination vs. DMSO. (I) GO and KEGG pathway analysis of DEGs in group of Combination vs. DMSO. Red dots indicate up‐regulated genes, and blue dots indicate downregulated genes. The *X*‐axis represents the gene ratio and the *Y*‐axis represents the GO and KEGG terms. The size of the circle indicates the enriched gene count and the color represents the −log10 (*p*‐value) of each term. PRI: PRIMA‐1^met^; Oxa: oxaliplatin, L‐OHP; Combi: combination of PRI and Oxa; GO: Gene Ontology; KEGG: Kyoto Encyclopedia of Genes and Genomes. (J) Immunoblotting assays of WNT/β‐catenin and Hippo pathways. HCT116 cells were treated with DMSO, PRI (40 μM), L‐OHP (1 μM) and their combination for 48 h. Subsequently, the cells were lysed and western blotting was performed with the indicated antibodies. β‐actin was used as a loading control.

Next, we explored the mechanisms of combined PRIMA‐1^met^ (PRI) and oxaliplatin (Oxa, L‐OHP) treatment on HCT116 (p53‐wt) and DLD‐1 (p53‐mutant) cell lines. The full list of all DEGs was included in Table S1. Our initial focus was on evaluating the individual cellular responses triggered by each drug. Analysis of DEGs in the p53‐wt HCT116 cell line, treated with L‐OHP, unveiled an increase in 381 genes and a decrease in 1296 genes compared to the DMSO controls (Figure 5A). The top 50 genes ranked by fold change were shown in Figure 5B. The upregulated genes demonstrated significant enrichment in extracellular matrix remodeling and cell adhesion, DNA damage, reactive oxygen species and p53 signaling pathways (Figure 5C), while the downregulated genes formed clusters related to cell division and cell cycle progression pathways, including chromosome segregation, cell‐cycle phase transition, and DNA damage repair (Figure 5C). These observations are consistent with L‐OHP's primary cytotoxic effect, which operates through interfering with the DNA replication and repair processes within cancer cell and influencing tumor microenvironment. PRIMA‐1^met^ treatment led to the upregulation of 645 genes associated with response to unfolded protein and endoplasmic reticulum (ER) stress pathways (Figure 5D–F). This reaction aligns with the established mechanism of PRIMA‐1^met^, which is activated in response to the accumulation of unfolded or misfolded proteins in the ER. Initially, it serves as a reparative response, but it escalates to apoptosis when the cell's capacity to restore homeostasis is exceeded. Additionally, a downregulation was noted in pathways vital to cancer progression, such as the Wnt signaling and Hippo pathway (Figure 5D–F). In HCT116 cells co‐treated with PRIMA‐1^met^, and L‐OHP, a more profound change in gene expression pattern, with 1902 downregulated genes and 1124 upregulated genes, was observed. The significant upregulation of genes related to oxidative stress, autophagy, mitophagy, ferroptosis, ER stress pathways was observed (Figure 5G–I), while the downregulated genes were enriched for Wnt signaling, gastric cancer, Hippo pathway, pathways regulating pluripotency of stem cell, DNA replication, and mismatch repair. The conserved Wnt/β‐catenin signaling has been broadly implicated in human cancers [38]. Phosphorylation of β‐catenin promotes its degradation via the ubiquitin/proteasome pathway [39]. YAP and TAZ are critical downstream effectors of the Hippo pathway that promotes contact‐dependent cell growth and proliferation in cancer cells. CTGF has been identified as YAP target gene [40]. Co‐treatment with the two drugs significantly increased the phosphorylation of β‐catenin and decreased YAP and CTGF protein levels (Figure 5J). Therefore, these data demonstrate that the co‐administration of PRIMA‐1^met^—L‐OHP inhibits both the Wnt and Hippo signaling pathways in HCT116 cells.

**FIGURE 6:**
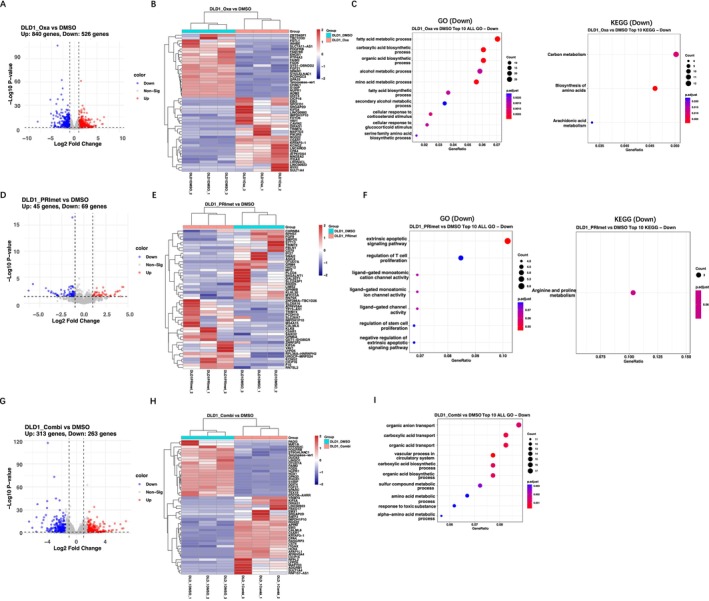
Analysis of DEGs in p53‐mutant DLD‐1 treated with different modalities. (A) Volcano plot of all significantly changed and (B) heatmap of top 50 DEGs ranked by fold change in L‐OHP vs. DMSO. (C) GO and KEGG pathway analysis of DEGs in group of L‐OHP vs. DMSO. (D) Volcano plot of all significantly changed and (E) heatmap of top 50 DEGs ranked by fold change in PRI vs. DMSO. (F) GO and KEGG pathway analysis of DEGs in group of PRI vs. DMSO. (G) Volcano plot of all significantly changed and (H) heatmap of DEGs screening between combination vs. DMSO. (I) GO term analysis of DEGs in group of combination vs. DMSO. Red dots indicate upregulated genes, and blue dots indicate down‐regulated genes. The *X*‐axis represents the gene ratio and the *Y*‐axis represents the GO and KEGG terms. The size of the circle indicates the enriched gene count and the color represents the −log10 (*p*‐value) of each term. PRI: PRIMA‐1^met^; Oxa: oxaliplatin, L‐OHP; Combi: combination of PRI and Oxa; GO: Gene Ontology; KEGG: Kyoto Encyclopedia of Genes and Genomes.

In the p53 mutant DLD‐1 cell line, L‐OHP induced increase in expression of 840 genes and decrease in expression of 526 genes relative to DMSO control (Figure 6A,B). The majority of enriched pathways in downregulation list were metabolism related, such as fat acid metabolic process, carboxylic acid biosynthetic process, carbon metabolism, and biosynthesis of amino acids (Figure 6C). Our transcriptomic analysis identified PRIMA‐1^met^ positively regulated 45 genes and negatively regulated 69 genes (Figure 6D,E). Noticeable changes in extrinsic apoptosis signaling pathways were observed, suggesting a potential revival of p53 activity (Figure 6F). Co‐treatment of PRIMA‐1^met^ and L‐OHP on DLD‐1 cells led to a downregulation of 263 genes linked to metabolic pathways, specifically those associated with carbohydrate and fatty acid metabolism (Figure 6G–I). This reflects an adaptive response to the stress and damage induced by the drug response.

In summary, our data indicate that the synergistic effects of PRIMA‐1^met^ and L‐OHP on CRC cells through both p53‐dependent and ‐independent modalities, such as intrinsic and extrinsic apoptotic pathways, cell cycle arrest, and relevant stress response characterized by alterations in redox balance, metabolic processes, and ER functionality. Moreover, inhibition of Wnt signaling and Hippo pathway may contribute to the synergistic effects of PRIMA‐1^met^ and L‐OHP on p53‐wt CRC cells.

### 3.5 Regulatory Network Construction and Module Detection by WGCNA

**FIGURE 7:**
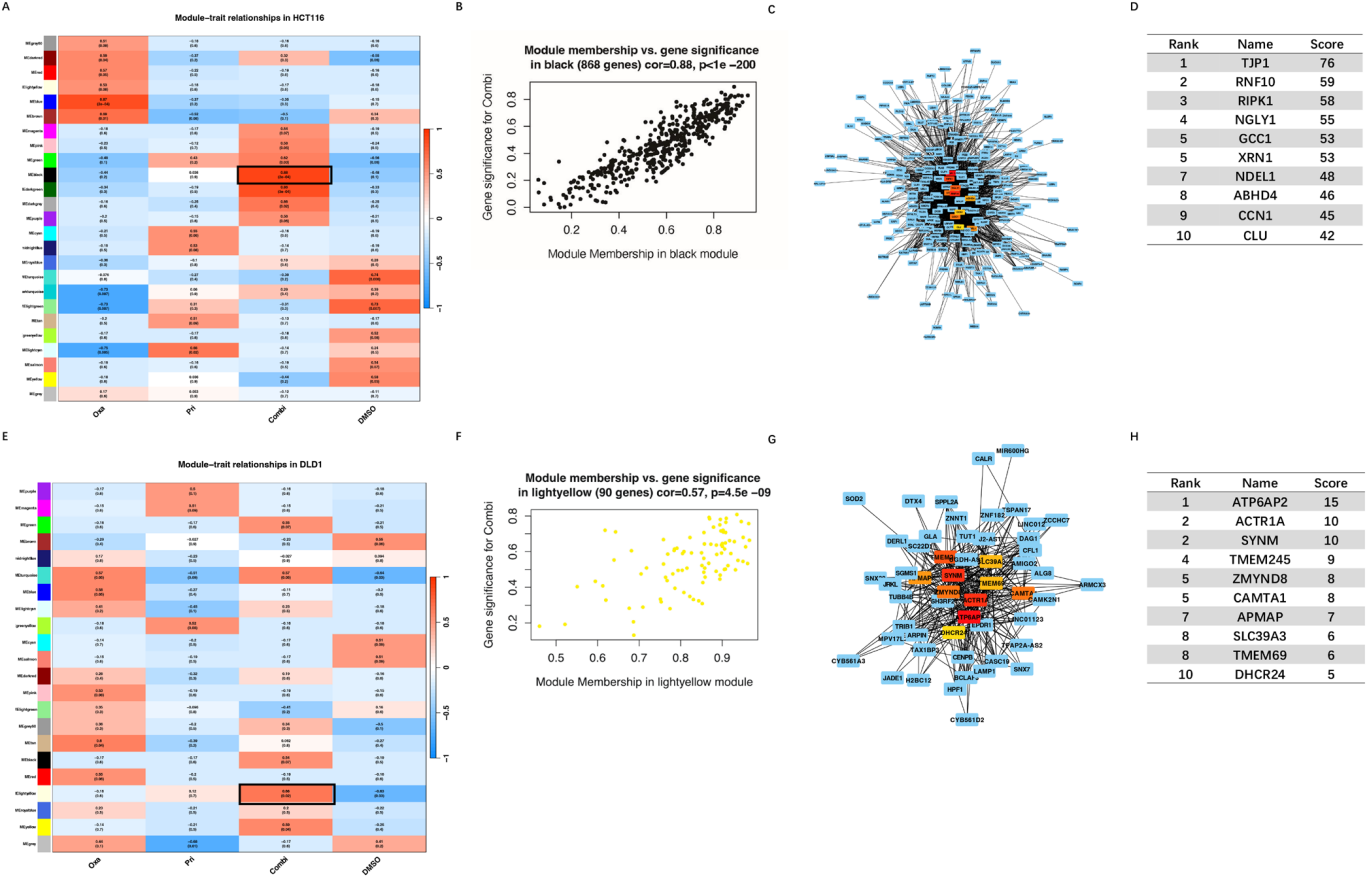
(A) Heatmap of correlation between the module eigengenes and treatment modalities of HCT116 cells. The MEblack‐grade block, highlighted by a black rectangle, was selected for subsequent analysis. (B) Correlation of black module membership and gene significance in combination treated HCT116 group. (C) Interaction of gene co‐expression patterns in the black module of combination treated HCT116 group. The module was visualized using Cytoscape_3.10.1 software. (D) The top 10 hub genes identified the CytoHubba algorithms based on degree in the black module of combination treated HCT116 group. (E) Heatmap of the correlation between the module eigengenes and treatment modalities of DLD‐1 cells. The MEligthyellow‐grade block, highlighted by a black rectangle, was selected for subsequent analysis. (F) Correlation of light‐yellow module membership and gene significance in combination treated DLD‐1 group. (G) Interaction of gene co‐expression patterns in the light‐yellow module of combination treated DLD‐1 group. (H) The top 10 hub genes identified based on degree in the light‐yellow module of combination treated DLD‐1 group. ME, module eigengene; GS: gene significance; PRI: PRIMA‐1^met^; Oxa: oxaliplatin, L‐OHP; Combi: combination of PRI and Oxa.

To unravel the key driving events behind the synergistic drug effect, we conducted WGCNA analysis assign genes with similar expression patterns into one module, and 25 modules were obtained in p53‐wt HCT116 cells treated with different modalities (Figure 7A). After screening for strong correlations between all modules and different treatment groups, we found the module eigengene (ME) in the black module exhibited a highest correlation with combination treatment than other modules (Figure 7B, *r* = 0.88 and *p* < 1E‐200). Hence, this black module, containing 868 genes, was selected as key module for further analysis and its scatter plots of GS vs. module membership was shown in the Figure 7B. We constructed gene co‐expression networks (Figure 7C), and pinned down the top 10 genes with the highest degree of connection, designating them as hub genes, including TJP1, RNF10, RIPK1, NGLY1, GCC1, XRN1, NDEL1, ABHD4, CCN1, CLU (Figure 7D). The hub genes TJP1 [41], RIPK1 [42], NGLY1 [43], XRN1 [44], NDEL1 [45], CCN1 [46], and CLU [47], play vital roles in tumorigenesis.

Similarly, WGCNA analysis revealed 22 modules identified in p53‐mutant DLD‐1 groups (Figure 7E). By assessing correlations between all modules and different treatment groups, we discovered that the ME in the light‐yellow module, comprising 90 genes, exhibited the highest correlation with combination treatment compared to other modules (Figure 7F, *r* = 0.66 and *p* = 4.5E‐09). Subsequently, we constructed gene co‐expression networks in the light‐yellow module (Figure 7G) and identified the top 10 genes with the highest degree of connection, designating them as hub genes, containing ATP6AP2, ACTR1A, SYNM, TMEM245, ZMYND8, CAMTA1, APMAP, SLC39A3, TMEM69, DHCR24 (Figure 7H). The hub genes ATP6AP2 [48], ACTR1A [49], SYNM [50], ZMYND8 [51], CAMTA1 [52], SLC39A3 [53], and DHCR24 [54], are associated with cancer development and progression.

### 3.6 Dual Administration of PRIMA‐1^met^ and L‐OHP Is an Effective Treatment for CRC in vivo

**FIGURE 8:**
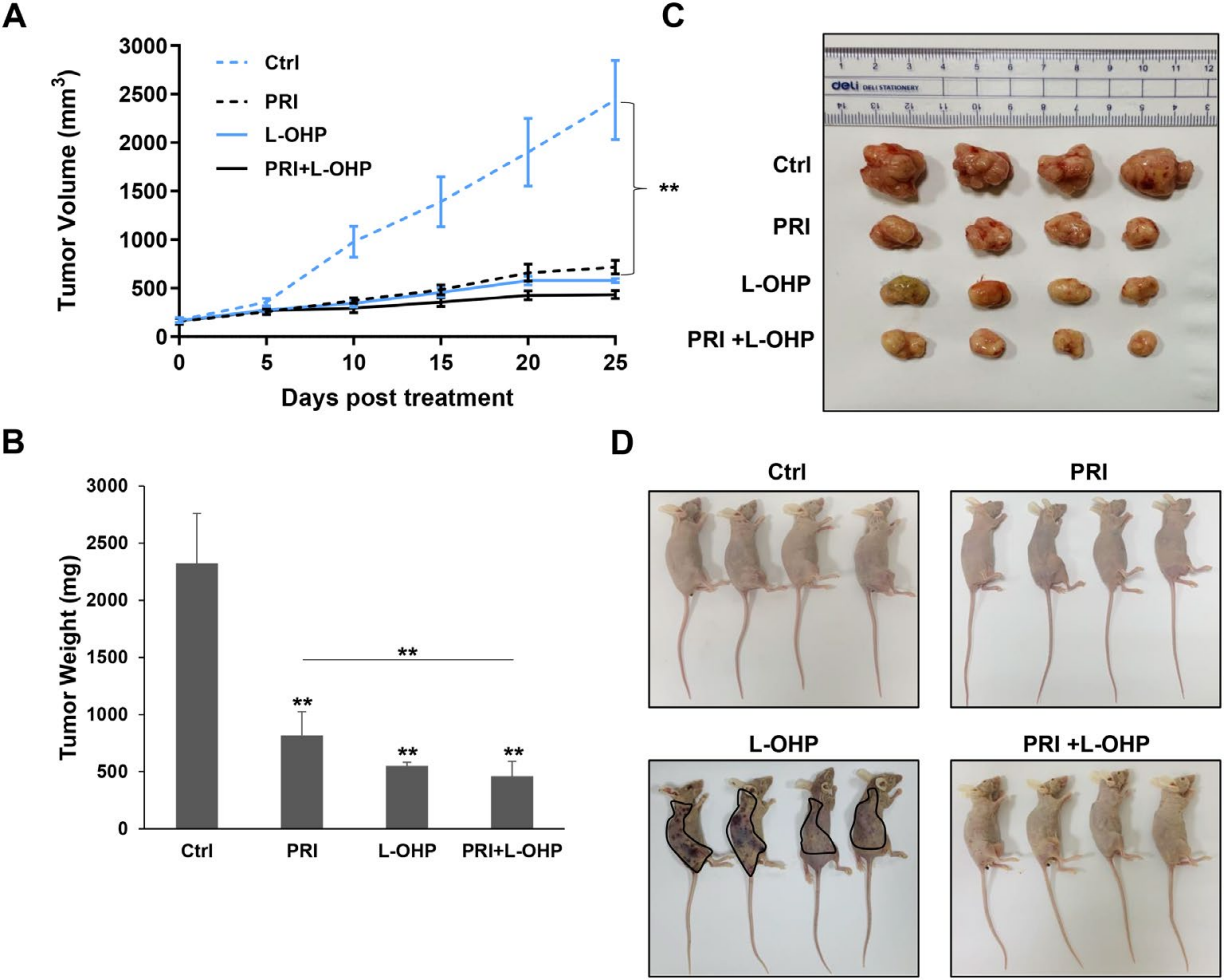
The combination of PRIMA‐1^met^ and L‐OHP efficiently suppressed CRC xenograft growth with low toxicity. (A) DLD‐1 (p53‐mutant) cells were inoculated into BALB/c mice (via subcutaneous injection) to establish a CRC tumor model. The detailed treatment regime and group were indicated in the Materials and Methods section. The tumor volume was measured by caliper. The tumor growth curves were constructed according to the average tumor volume of each group ± SD (mm^3^). **p < 0.01. (B) At the end of experiments, mice were sacrificed, and then the tumor weight was taken as shown in the bar graph. Data were reported as the mean ± SD and were analyzed by Student's *t*‐test; *n* = 4 mice per group. **p < 0.01. (C) Images of DLD‐1 tumor xenografts from mice treated with vehicle control and different drugs after dissection were shown. (D) Images of whole bodies of xenograft mice treated with vehicle control and different drugs were displayed. Petechiae and ecchymoses caused by abnormal clotting and bleeding were presented in L‐OHP‐treated mice (indicated by black shapes), but not in combination‐treated mice. Ctrl: vehicle control; PRI: PRIMA‐1^met^; L‐OHP: oxaliplatin.

Based on the above‐mentioned in vitro results, the in vivo efficacy of the combination regimen was evaluated in nude mice bearing CRC tumors induced by DLD‐1 cells. Both of the monotherapies and combined treatment significantly impeded tumor growth compared to the vehicle control by the 10th day (*p* < 0.01, Figure 8A). By Day 25 post‐treatment, all the 3 treatment groups exhibited significantly lower tumor weights than the control group (*p* < 0.01). Furthermore, the combined therapy led to a notable additional reduction in tumor weight compared to the administration of PRIMA‐1^met^ alone (*p* < 0.01, Figure 8B,C).

In contrast, there were no discernible differences in the appearance, daily activities, and eating behavior among the vehicle control group and the PRIMA‐1^met^‐treated or combination therapy groups. This suggests that the concurrent administration of PRIMA‐1^met^ and L‐OHP did not increase overall toxicity in the mice. Interestingly, petechiae and ecchymoses related to abnormal clotting and bleeding appeared over the body of L‐OHP‐treated mice (Figure 8D) [55, 56], and these mice were skinnier than mice in the other treatment groups (Figure 8D), which are the signs of L‐OHP‐caused side effects. Notably, these adverse effects caused by L‐OHP were reduced in mice co‐treated with PRIMA‐1^met^ and L‐OHP simultaneously (Figure 8D). In summary, these results demonstrated that addition of PRIMA‐1^met^ with L‐OHP not only had a superior effect on inhibiting CRC xenograft tumor growth compared to either agent alone, but also helped alleviate L‐OHP‐related toxicity.

## 4 Discussion

In our investigation, we observed a heightened resistance to L‐OHP in p53‐mutant cell lines, specifically HCT15, SW620 and DLD‐1, in comparison to cell lines with p53‐wt. These results are consistent with prior research [57, 58], indicating that the presence of mutant p53 may lead to reduced sensitivity to L‐OHP in CRC patients. Unfortunately, the dismal fact is that p53 mutation occurs in approximately 40%–50% of CRC cases. Consequently, there is an urgent need to devise innovative strategies to overcome the resistance exhibited by CRC cells to L‐OHP [15].

The majority of p53 mutations are missense mutations concentrated in the DNA‐binding core domain, resulting in “contact” or “structural” alterations to the protein. These changes invariably lead to the loss or impairment of its DNA‐binding properties [59]. Despite this, the apoptotic pathway downstream of p53 is likely to remain intact in CRC cells carrying mutant p53 [15]. Therefore, several mutant p53 reactivators have entered into clinical trials for hematologic malignancies and solid tumors with mutant p53 for precision anticancer medicine [19, 60]. A notable phase Ib/II study showcased that the combination of PRIMA‐1^met^ and azacytidine achieved a high rate of clinical response and molecular remissions in patients with p53 mutant myelodysplastic syndromes (MDS) or oligoblastic AML [61]. Another phase I study involving a three‐drug combination of PRIMA‐1^met^ with venetoclax and azacitidine in p53‐mutant AML patients demonstrated an acceptable safety profile and encouraging clinical response [62]. A number of pre‐clinical studies have demonstrated that PRIMA‐1^met^ synergizes with cisplatin in ovarian cancer [27], with 5‐FU in esophageal adenocarcinoma [28], as well as with radiotherapy [63] and targeted therapy [64, 65]. These encouraging pre‐clinical and clinical evidence led us to investigate the effect of PRIMA‐1^met^ combined with L‐OHP on CRC both in vitro and in vivo, aiming to mitigate L‐OHP resistance and alleviate L‐OHP‐related toxicity. Several different cell lines across various assays was utilized to minimize cell line‐specific effects, thereby making our findings more broadly applicable. Our results unveiled that the heightened cytotoxicity in CRC cells stemmed from a synergistic interplay between the two drugs, and this effect was not contingent on the p53 status. The strongest synergism between PRIMA‐1^met^ and L‐OHP was observed in all the three p53‐mutant cell lines, suggesting the drug combination achieves greater efficacy in p53‐mutant CRC cells. Additionally, we noted a significantly robust inhibition of cell migration, invasion, and colony formation in CRC cells treated simultaneously with both drugs, whether expressing wt p53 or mutant p53, in comparison to the effect of either drug administered alone.

In order to provide novel insight into our understanding of the molecular mechanism of different drugs in treating colon cancer, RNA‐seq data from a p53‐wt HCT116 and p53‐mutant DLD‐1 CRC cells treated with PRIMA‐1^met^, L‐OHP, and PRIMA‐1^met^ plus L‐OHP were further analyzed. Overall, the numbers of DEGs in either single agent groups or combination groups of HCT116 were greater than those of DLD‐1 cells. These differential responses could be explained by the fact that mutant p53 in DLD‐1 cells confer certain degree of resistance to various drug treatment. In combination treated HCT116 group, the top pathways altered concentrated on autophagy, oxidative stress, unfold protein response, ER stress, Wnt and Hippo pathways. While in DLD‐1 cells, combination treatment led to changes in metabolism and transport related pathways. The Wnt and Hippo signaling pathways are pivotal in maintaining tissue homeostasis and organ size, regulating cell proliferation, differentiation, and apoptosis [66, 67, 68, 69]. Dysregulation of these pathways is a common occurrence and collaborative to promote tumorigenesis in multiple cancers, including CRC [70, 71]. Therefore, the simultaneously targeting these intertwined roles and overlapping functions caused by these altered signaling pathways contribute to the robust and synergistic anti‐CRC effects observed in combination of PRIMA‐1^met^ and L‐OHP.

We applied WGCNA to identify key modules and hub genes related to combination therapy in these two types of CRC cells. The black module was most positively correlated with PRIMA‐1^met^‐L‐OHP treatment in p53‐wt HCT116. Among the top 10 hub genes, TPJ1 involves in the formation and maintenance of tight junctions in epithelial cells and tight junction proteins contribute to cancer cell proliferation, transformation and metastasis [41]. RIPK1 is a crucial regulator of cell death and inflammation, playing an important role in CRC cells resistance to necroptosis [37, 72]. The exonuclease XRN1 has recently been identified as a novel target for cancer immunotherapy [44]. CLU is well‐known to promote tumor survival and resistance to therapy in multiple cancers, including CRC [46, 73]. The light‐yellow module exhibited the highest positive correlation with PRIMA‐1^met^ plus L‐OHP treatment in p53‐mutant DLD‐1 cells. One of the top 10 hub genes, ATP6AP2, encoding an adenosine triphosphatases (ATPases), facilitates CRC through activation of the Wnt signaling pathway [48]. Additionally, ACTR1A is involved in microtubule‐based vesicle motility and chromosome segregation [49]. SYNM is an important plasma marker of recurrent glioblastoma [50]. Both ZMYND8 and DHCR24 promote breast cancer stem cell survival [51, 54]. Overall, these hub genes in black and light‐yellow modules have been widely implicated in cancer survival, recurrence and drug resistance. It is worth noting that these two modules emphasize gene co‐expression and these hub genes may not necessarily have direct or indirect interactions. They provide an additional biological dimension into the combination therapy of PRIMA‐1^met^ and L‐OHP, complementing the insights gained from GO enrichment and KEGG pathway analyses.

One notable finding in our in vivo study is that combined PRIMA‐1^met^ with L‐OHP not only exerted a stronger anti‐tumor effect than PRIMA‐1^met^ monotherapy, but also showed less toxicity in mice than L‐OHP alone, which has not been reported before our study. It is worth noting that L‐OHP‐induced hematologic toxicity is a severe side effect, including anemia, neutropenia and thrombocytopenia [55, 56, 74]. Some CRC patients have had grievous or even fatal toxicity because of acute‐onset thrombocytopenia, haemolysis and bleeding caused by L‐OHP‐based treatment [56, 75]. Crucially, the incorporation of PRIMA‐1^met^ mitigates the toxicity inflicted on hematopoiesis by L‐OHP. This holds significant and practical clinical implications, offering a triple‐fold advantage of enhanced therapeutic effects, reduced L‐OHP resistance, and prevention of L‐OHP‐related side effects for patients.

## 5 Conclusions

Therefore, our study shows that combining PRIMA‐1^met^ with L‐OHP is a promising strategy for overcoming L‐OHP resistance in CRC, particularly in p53‐mutant cells. The combination exhibited strong synergistic effects, significantly inhibiting cell migration, invasion, and colony formation, irrespective of p53 status.

Transcriptomic analysis revealed key pathways and hub genes associated with the response, offering new insights into the molecular mechanisms driving the observed synergy. Notably, the combination therapy not only enhanced antitumor efficacy but also reduced L‐OHP‐related toxicity in vivo, particularly hematologic side effects. These findings provide compelling justification of this combination therapy for improving treatment outcomes and reducing side effects in CRC patients and warrant further clinical investigation.

## Author Contributions

**Xiao‐lan Li:** conceptualization (equal), data curation (equal), formal analysis (equal), funding acquisition (equal), investigation (equal), methodology (equal), resources (equal), writing – original draft (equal). **Jianbiao Zhou:** conceptualization (equal), data curation (equal), formal analysis (equal), investigation (equal), methodology (equal), writing – original draft (equal). **Nicole Xin‐Ning Tang:** formal analysis (supporting), investigation (supporting), methodology (supporting). **Yi Chai:** formal analysis (supporting), investigation (supporting), methodology (supporting). **Meng Zhou:** formal analysis (supporting), investigation (supporting), methodology (supporting). **Ai‐di Gao:** formal analysis (supporting), investigation (supporting), methodology (supporting). **Zhong‐kai Lu:** conceptualization (equal), data curation (equal), formal analysis (equal), funding acquisition (equal), investigation (equal), methodology (equal), resources (equal). **Han Min:** conceptualization (lead), formal analysis (equal), funding acquisition (equal), investigation (equal), methodology (equal), resources (equal), supervision (equal), writing – original draft (equal).

## Ethics Statement

The animal study was approved by the Animal Ethics Committee of Nanjing Medical University (protocol number: 2018‐036).

## Consent

All author reviewed the final manuscript and consented for publication.

## Conflicts of Interest

The authors declare no conflicts of interest.

## Supporting information


Table S1.

## Data Availability

The RNA‐seq data have been deposited into the Gene Expression Omnibus (GEO) repository with the accession number GSE254323. All data relevant to the study are included in the article.
